# Risk Factors for Flap Loss: Analysis of Donor and Recipient Vessel Morphology in Patients Undergoing Microvascular Head and Neck Reconstructions

**DOI:** 10.3390/jcm12165206

**Published:** 2023-08-10

**Authors:** Johannes G. Schuderer, Huong T. Dinh, Steffen Spoerl, Jürgen Taxis, Mathias Fiedler, Josef M. Gottsauner, Michael Maurer, Torsten E. Reichert, Johannes K. Meier, Florian Weber, Tobias Ettl

**Affiliations:** 1 Department of Oral and Maxillofacial Surgery, University Hospital Regensburg, 93053 Regensburg, Germanyjuergen.taxis@ukr.de (J.T.); mathias1.fiedler@ukr.de (M.F.);; 2 Institute of Pathology, University Hospital Regensburg, 93053 Regensburg, Germany

**Keywords:** microvascular reconstruction, free flap, flap loss, vessel anatomy, anastomosis

## Abstract

In microvascular head and neck reconstruction, various factors such as diabetes, alcohol consumption, and preoperative radiation hold a risk for flap loss. The primary objective of this study was to examine the vessel morphology of both recipient and donor vessels and to identify predictors for changes in the diameters of H.E.-stained specimens associated with flap loss in a prospective setting. Artery and vein samples (N = 191) were collected from patients (N = 100), with sampling from the recipient vessels in the neck area and the donor vessels prior to anastomosis. External vessel diameter transverse (ED), inner vessel diameter transverse (ID), thickness vessel intima (TI), thickness vessel media (TM), thickness vessel wall (TVW), and intima-media ratio (IMR) for the recipient (R) and transplant site (T) in arteries (A) and veins (V) were evaluated using H.E. staining. Flap loss (3%) was associated with increased ARED (*p* = 0.004) and ARID (*p* = 0.004). Preoperative radiotherapy led to a significant reduction in the outer diameter of the recipient vein in the neck (*p* = 0.018). Alcohol consumption (*p* = 0.05), previous thrombosis (*p* = 0.007), and diabetes (*p* = 0.002) were associated with an increase in the total thickness of venous recipient veins in the neck. Diabetes was also found to be associated with dilation of the venous media in the neck vessels (*p* = 0.007). The presence of cardiovascular disease (CVD) was associated with reduced intimal thickness (*p* = 0.016) and increased total venous vessel wall thickness (*p* = 0.017) at the transplant site. Revision surgeries were linked to increased internal and external diameters of the graft artery (*p* = 0.04 and *p* = 0.003, respectively), while patients with flap loss showed significantly increased artery diameters (*p* = 0.004). At the transplant site, alcohol influenced the enlargement of arm artery diameters (*p* = 0.03) and the intima–media ratio in the radial forearm flap (*p* = 0.013). In the anterolateral thigh, CVD significantly increased the intimal thickness and the intima–media ratio of the graft artery (*p* = 0.01 and *p* = 0.02, respectively). Patients with myocardial infarction displayed increased thickness in the *A. thyroidea* and artery media (*p* = 0.003). Facial arteries exhibited larger total vessel diameters in patients with CVD (*p* = 0.03), while facial arteries in patients with previous thrombosis had larger diameters and thicker media (*p* = 0.01). The presence of diabetes was associated with a reduced intima–media ratio (*p* < 0.001). Although the presence of diabetes, irradiation, and cardiovascular disease causes changes in vessel thickness in connecting vessels, these alterations did not adversely affect the overall success of the flap.

## 1. Introduction

Microvascular surgery is an established standard therapy for the functional rehabilitation of patients with defects in the head and neck region [1,2]. Microvascular grafts, such as the radial forearm flap (RFF) and the free fibula flap (FFF), facilitate the reconstruction of intricate defect scenarios by replacing multiple tissues in a single approach. Moreover, these grafts offer surgeons a sufficiently long vascular pedicle with a substantial vessel diameter [3,4]. Despite very good overall success rates of 95%, there are well-known factors that hold a risk of flap loss and seem to influence overall patient outcomes by compromising arterial and venous perfusion [5]. Smoking, alcohol consumption, and atherosclerosis have been shown to impact the success of microvascular reconstruction inducing histomorphologically apparent detrimental effects on vessels by causing endothelial dysfunction, chronic inflammation, and oxidative stress [6,7,8,9]. One additional common factor that seems to predict therapy setbacks is preoperative irradiation [5,10,11].

Upon histopathological examination, the morphology of free flap donor and recipient vessels in patients at risk show increased microscopic changes toward hyalinosis and inflammatory or prothrombotic features [12,13]. Next to changes in vessel wall diameters, changes in intima and media thickness in affected vessels are also presumed [14,15,16]. In addition, especially individuals with diabetes and arteriosclerosis may exhibit reduced vascular compliance, arterial stiffness, and impaired endothelial function, all of which can further impact the success of microvascular reconstruction by reducing local blood flow [17,18].

In this prospective study, we aimed to examine vessel morphology in both recipient and donor vessels and to identify predictors for changes in the diameters of H.E.-stained specimens. This may provide valuable insights into the impact of epidemiological factors on the success of microvascular reconstruction.

## 2. Material and Methods

All patients included in this study underwent ablative surgery and microvascular reconstruction due to neoplastic (tumor) or inflammatory diseases (osteomyelitis, necrosis) in the maxillofacial area at the Department of Oral and Maxillofacial Surgery. Both artery and vein samples were collected from the patient before anastomosis, with sampling from the recipient vessels in the neck area prior to suturing the graft. Samples of the donor vessels were taken from the pedicle immediately after graft harvest. Only vessels with intact integrity of the intima, media, and adventitia were submitted to pathology, while any vessels that were damaged or torn were excluded from the analysis.

The vascular specimens were fixed in 4% neutral buffered formalin in the operating theatre and transferred to the Institute of Pathology for complete formaldehyde fixation. Paraffin wax blocks were prepared using “ASP300S” (Leica Biosystems, Richmond, IL, USA) and “Histo Star” (Thermo Fisher Scientific, Schwerte, Germany). After cooling on the “PARA COOLER A” plate, the blocks were sectioned using the “Microm HM 340E with STS (Section Transfer System, Fisher Scientific, Schwerte, Germany)” rotary microtome and were mounted onto printed slides. The slides were then subjected to hematoxylin and eosin (HE) staining using “Histo Core SPECTRA ST” (Leica Biosystems, Richmond, IL, USA). All slides were digitally scanned using the Sysmex model “Panoramic 250 Flash III” (Sysmex, Norderstedt, Germany), and “Case Viewer” version 2.4 from the company 3D HISTECH Ltd (Budapest, Hungary). was used for microscopy and measurements.

Specific parameters were pre-defined to ensure consistency and reproducibility in measuring vessel diameter and stenosis in H.E. staining. The decision to measure these diameters was guided by the aim to maintain a straightforward examination under the microscope, encompassing external diameter, inner diameter, media and intima thickness, and total vessel wall thickness. For thickness and diameter measurements, a representative area of each vessel was carefully selected, excluding tangentially or only partially sectioned areas. A prior calibration of the measurement tool was conducted, and then the digital measurement tool was applied. Vessel examination was performed using a standardized 40× magnification by a specialist in clinical pathology.

## 3. Patient Data

The records of patients who received microvascular flap reconstruction in this study were filtered. The patient data were evaluated, and a descriptive analysis was performed regarding epidemiological data, preoperative radiotherapy, nicotine and alcohol abuse, cardiovascular disease, and length of stay. Perioperative diagnoses were only included if they were ICD encoded in the discharge letter. In addition, tumor diagnosis or infectious states were recorded due to their ICD coding. With regard to microvascular reconstruction flap type, success and need for revision were documented.

From a prospective standpoint, flap success and flap revision were used as primary endpoints. In addition, the influence of the above-mentioned parameters on vessel wall thickness in H.E. staining was analyzed.

## 4. Statistics

Descriptive analyses were conducted in SPSS, and the variables were presented in absolute numbers and percentages. Univariate analyses were used to assess differences and correlations among the variables. The chi-squared test and *t*-test were used depending on the scale level and normal distribution of the compared variables. Statistical significance was set at *p* < 0.05. Vessel diameters were quantified in micrometers as the metric measurement unit. All analyses were conducted using SPSS version 26.0 (IBM Corp., Endicott, NY, USA).

## 5. Results

In this prospective study, we included 100 patients who received a microvascular graft for reconstruction in the head and neck region between 2021 and 2022.

The 100 patients consisted of 75 men and 25 women with a mean age of 65 ± 11.1 years. The patient population was divided into various diagnoses, including 63 oral squamous cell carcinomas, 17 cases of osteonecrosis of the jaw, 6 cases of osteomyelitis, and 6 cases of extraoral skin tumors. Eight patients underwent surgery and reconstruction for reasons such as trauma or salivary gland carcinomas. (Table 1).

Reconstruction was performed using various types of transplants, with free fibula flaps being the most frequently used (39%) followed by radial forearm flaps (37%). The mandible and floor of the mouth were the primary locations for reconstruction, accounting for 34% and 24% of cases, respectively. Other locations included the upper jaw (14%), tongue (9%), inner cheek (5%), and palate (5%). Nine cases required extraoral reconstruction, such as for the rehabilitation of the scalp after spinalioma resection.

The microvascular graft required revision in 6% of cases, and the overall success rate was 97%. The mean surgical time was 392 ± 104.2 min, with patients being hospitalized in the intensive care unit for an average of 4 ± 2.7 days and on the normal ward for 19 ± 9 days.

In terms of intraoperative vessels for microvascular anastomosis, the facial artery was selected for arterial anastomosis in 58% of cases, followed by the superficial thyroid artery (31%), the lingual artery (7%), and the superficial temporal artery (4%). For venous anastomosis, the facial vein was used in 47.3% of cases, the superficial thyroid vein in 35.5% of cases, the intrajugular vein in 10.7% of cases, and the external vein in 7.5% of cases. The superficial temporal vein was connected a total of four times.

In the retrospective patient evaluation, 66% of the patients had previously undergone head and neck radiotherapy, while 36% were documented to have nicotine abuse and 25% had alcohol abuse. A total of 10% of the patients had one or more thromboses prior to surgery, while 13% had experienced a myocardial infarction. In 27% of cases, cardiovascular disease was documented in the diagnoses of diabetes (14%). Detailed information is provided in Table 1.

The evaluation of vessel diameters using H.E. stain was conducted on a total of 70 transplant site arteries, 78 recipient site arteries, 13 transplant site veins, and 30 recipient site veins. Detailed results are provided in Table 2.

The univariate analyses showed that patients who received preoperative radiotherapy had a significant reduction in the outer diameter of the recipient vein in the neck (1966 µm vs. 2494 µm, respectively, *p* = 0.018). In addition, the total thickness of the venous recipient veins in the neck appeared to increase due to the influence of alcohol (519 µm vs. 360 µm, *p* = 0.05), previous thrombosis (505 µm vs. 389 µm, *p* = 0.007) and diabetes (476 µm vs. 396 µm, *p* = 0.002). The absolute thickness of the venous media in the neck vessels was significantly dilatated in the presence of diabetes (580 µm vs. 369 µm, *p* = 0.007). In addition, the presence of CVD led to a reduction in intimal thickness (1355 µm vs. 1613 µm, *p* = 0.016) and increased total venous vessel wall thickness (396 µm vs. 367 µm, *p* = 0.017) at the transplant site. A revision was significantly associated with an increased internal diameter of the graft artery (2018 µm vs. 1436 µm, *p* = 0.04) and increased external artery diameter at the neck (2667 µm vs. 2119 µm, *p* = 0.003). Patients with flap loss showed significantly increased vessel artery inner and outer diameter at the neck (3161 µm vs. 2120 µm, *p* = 0.004 resp. 2012 µm vs. 1188 µm, *p* = 0.004).

Breaking down the analyses by transplant revealed a significant enlargement in the outer diameter of arm arteries (2946 µm vs. 2604 µm, *p* = 0.03) and inner diameter (1690 µm vs. 1482 m, *p* = 0.04) under the influence of alcohol and an enlargement of the intima–media ratio of the vein in the RFF (0.14 vs. 0.09, *p* = 0.013). In ALT, CVD was shown to increase the intimal thickness (169 µm vs. 110 µm, *p* = 0.01) and the intima–media ratio of the graft artery (0.31 vs 0.30, *p* = 0.02) significantly. Fibula transplants were evaluated but did not show any association with the clinical parameters (Table 3).

Univariate analyses also showed clinical differences in the individual vascular parameters regarding vessel type. In patients with myocardial infarction, the *A. thyroidea* as a recipient vessel showed an increase in the absolute vessel thickness (925 µm vs. 535 µm, *p* = 0.003) and an increase in the artery media thickness (701 µm vs. 413 µm, *p* = 0.003). Larger total vessel diameters (584 µm vs. 511 µm, *p* = 0.03) were measured for the facial artery in patients with CVD. Recipient facial arteries from patients with previous thrombosis were also larger (672 µm vs. 511 µm, *p* = 0.01) and had a thicker media (512 µm vs. 395 µm, *p* = 0.01). Finally, specimens with the presence of diabetes had a significantly reduced intima–media ratio (0.13 vs. 0.32, *p* < 0.001).

**Figure 1:**
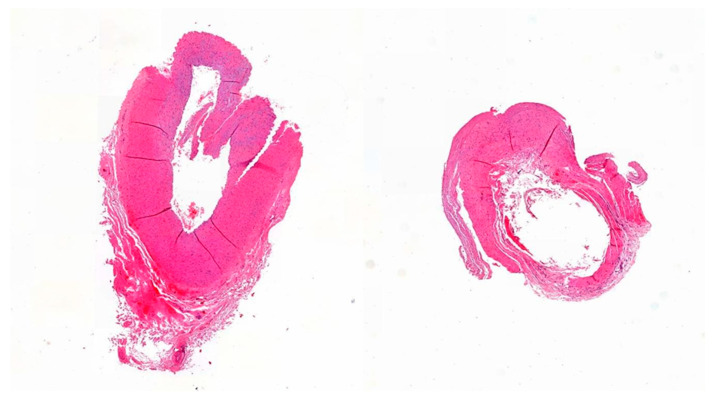
Recipient temporal superficial artery (**left**) without and (**right**) with pre radiotherapy H.E. staining, 40× zoom.

In terms of radiation, the *A. facialis* showed a significantly lower intern diameter (1246 µm vs 1149 µm, *p* = 0.04) and a smaller intima (116 µm vs. 144.5 µm, *p* = 0.01) with a reduced intima–media ratio (0.3 vs 0.32, *p* = 0.02). The temporal artery showed significantly lower total vessel wall thickness (296.6 µm vs. 876 µm, *p* = 0.04) and reduced media thickness (235.5 µm vs. 790 µm, *p* = 0.02). The IMR was increased (0.24 vs 0.11, *p* = 0.01) (Figure 1).

In the case of facial venous connecting vessels, alcohol had an influence on the thickness of the vein (472 µm vs. 388 µm, *p* = 0.038) and the media thickness (452 µm vs. 366 µm, *p* = 0.035). Diabetes increased vessel thickness (592 µm vs. 388 µm, *p* = 0.027) and media thickness (577 µm vs. 366 µm, *p* = 0.022), respectively. In addition, the intima–media ratio appeared to be reduced (0.02 vs 0.07, *p* < 0.001). Regarding the internal jugular vein, nicotine (20 µm vs. 15 µm, *p* = 0.002) and CVD (12 µm vs. 15 µm, *p* = 0.002) each lead to a reciprocal change in intimal thickness (Table 4).

## 6. Discussion

In general, the reconstruction of head and neck defects using free microvascular transplants represents an essential aspect of routine clinical practice. The growing proportion of older and medically complex patients presents clinical challenges during the procedural planning phase. Accurate preoperative visualization of vessels is critical, especially in flap preparation, as observed in the case of free fibula flap (FFF). However, challenges during anastomosis unrelated to the flap’s macroscopic characteristics may emerge, potentially resulting in immediate revision or flap loss. [19]. Pries and colleagues demonstrated the influence of both local and systemic stress on the adaptive capacity of peripheral and central vessels, revealing that vessel wall thickness adapts to both mechanical and metabolic stimuli [20].

In our investigation, we examined the impact of diverse patient-related factors on the morphology of both the donor and recipient vessels in H.E. staining and their correlation with the outcome of flap success or revision.

In general, the need for transplant revision or flap loss appears to be multifactorial. One important factor is vessel quality and morphology during anastomosis. Traditionally, thrombosis in the vein or artery leads to congestion or reduced blood flow, manifested as a discoloration of the transplant and poor intraoperative perfusion. In our study, a total of 6% of the flaps were revised with an overall success of 97%. This is consistent with data on success rates in the literature [5,11].

An important factor that has been subject to controversial discussions in the literature is the impact of radiation on the vascular morphology of neck vessels, directly influencing the success of graft procedures. [10]. In a comprehensive study involving over 850 participants, Tan et al. failed to demonstrate any significant effect of preoperative irradiation on the success of microvascular reconstruction [21]. However, in a meta-analysis conducted by Mijiti et al., a pooled odds ratio (OR) of 1.82 was reported for flap loss in association with preoperative irradiation. [10].

In our study, the presence of preoperative irradiation was significantly associated with a reduction in the thickness of the recipient vein (*p* = 0.018) but was not associated with overall flap success. From a clinical point of view, this result corresponds to the increased risk of venous injury during the preparation of the venous recipient vessel. In further subgroup analyses, a significant reduction in the intima and media thickness was observed in *A.* and *V. facialis* and *A. temporalis*. (Table 3, Figure 1). In the context of the calvaria and lower jaw, the development of osteoradionecrosis (IORN) in the cranial and mandible vault following radiation therapy for local tumor control is not uncommon [22,23]. Subsequently, the connection of microvascular grafts via the temporal or facial vascular axis becomes necessary. However, flap success in the pre-irradiated area poses a significant challenge [24]. Shonka and colleagues conducted a study involving 62 microvascular scalp reconstructions, revealing that 89% of the reported complications occurred specifically within the pre-irradiated tissue region [25]. In their study, Hirsch et al. reported a marginal decrease in the flap success rate of 88% among patients undergoing mandibular reconstruction for osteoradionecrosis. Nevertheless, no statistically significant disparities were observed when compared to the primary tumor reconstruction group [26].

Preidl et al. explicated the mechanisms underlying vascular changes subsequent to radiotherapy in patients, unveiling the emergence of prothrombotic and inflammatory alterations that precipitate endothelial dysfunction [14]. It is plausible to posit that common factors, such as irradiation and high blood pressure, can reduce vascular vasodilation, which in turn disrupts the balance between the pro- and antithrombotic activity of the endothelium, as reported by Rajendran et al. in 2013 [27]. Despite this, microvascular reconstruction appears to be a safe and feasible option for patients with osteoradionecrosis of the jaw or scalp, with no significant decrease in success rates, according to a study by Sweeny et al. in 2021 [23].

Patients who underwent revision exhibited a significant increase in inner (ATID, *p* = 0.04) and external (ARED, *p* = 0.03) vessel diameter of the transplant artery upon microscopic examination. Moreover, the presence of flap loss was associated with a significant increase in the outer and inner diameters of the recipient neck arteries compared to the rest of the patient population (*p* = 0.004). These findings should be considered in the context of the overall results. Notably, alcohol abuse, a history of thrombosis, cardiovascular disease (CVD), and diabetes were all associated with increased thickness of vessel segments. In particular, a direct correlation between these factors and an increase in overall vessel wall thickness of recipient veins in the neck was observed (*p* = 0.03) (see Table 5).

The presence of diabetes was associated with a significant increase in venous media enlargement (*p* = 0.007). Moreover, the analysis based on adjacent vessels revealed a noteworthy decrease in the intima-to-media ratio for both the facial artery (ARIMR: *p* < 0.001) and facial vein (VRIMR: *p* < 0.001) at the anastomosis site. According to Ueno et al. (2021), vessel wall thickness increases in patients with diabetes, which ultimately leads to impaired arterial blood flow [16]. The mechanisms underlying this phenomenon include hypertension with initial hyperperfusion and subsequent endothelial dysfunction, resulting in the expression of endothelin-1 and angiotensin II, ultimately leading to the remodeling of vascular anatomy with hypertrophy and fibrosis [28,29]. Valentini et al. were able to describe diabetes from various risk factors as a clear independent predictor for a worse flap outcome [30].

Moreover, there is a noticeable association between thrombosis and an increase in wall and media thickness in both the arteries and veins located in the neck (*p* = 0.007, *p* = 0.016, *p* = 0.024). The plausibility of the relationship between a history of thrombosis and changes in the morphology of vessels in the extremities or neck is evident. As per Falanga et al., cancer patients exhibit an imbalance in the hemostatic system that makes them up to seven times more vulnerable to thrombosis [31]. This hypercoagulable state arises from both direct and indirect mechanisms, resulting in the formation of thrombi [32]. Furthermore, there is evidence to suggest a long-term alteration of the patient’s vessels through the expression of metalloproteinases and subsequent vascular remodeling [33]. However, it is important to note that the risk of developing thrombosis and changes in vessel morphology in the extremities and neck share similar risk factors, including age, diabetes, and cardiovascular disease, as well as prior chemotherapy, radiation therapy, or medication use [32].

Our analysis suggests a discernible impact of long-term alcohol abuse on both the flap and neck vessel sites. Specifically, the outer diameter of the arterial vessel (*p* = 0.05) and the media (*p* = 0.03) flap site appeared to be significantly thickened (*p* = 0.005). Moreover, the entire vessel wall of the recipient vein was observed to be thickened as well (*p* = 0.05). Remarkably, in the sub-analysis, the same effects in the RFF (*p* = 0.03) and facial vein (*p* = 0.03) were observed. The influence of long-term alcohol consumption on vascular anatomy is multifaceted, with ethanol exerting both vascular and central effects on various regulatory axes, such as intracellular calcium levels and NO regulation, which may modulate vasodilation [34,35]. Additionally, the renin–angiotensin–aldosterone system (RAAS) is involved, resulting in elevated blood pressure [34], which together finally leads to atherosclerosis [36]. Hence, all the factors mentioned above not only contribute to the deterioration of the patient’s overall health, thereby elevating the risk of postoperative nosocomial complications, but also directly induce visible alterations in the vessels. These changes may pose challenges for surgeons during anastomosis, even under optimal conditions. Therefore, it becomes imperative to acknowledge and address these factors proactively for better surgical outcomes in the future.

This study has certain limitations. Despite its prospective design, the histopathologic examination of vessel gating may be susceptible to potential errors. Alongside the possibility of erroneous staining, there exists a potential concern that the chosen sections might not be entirely representative, thereby potentially leading to over- or underestimation of the corresponding vessel diameters. To bolster these aspects, the incorporation of immunohistochemical techniques could provide additional support and reliability to the findings.

## 7. Conclusions

In conclusion, our study revealed significant associations between vascular parameters in the context of flap loss, preoperative radiotherapy, alcohol consumption, previous thrombosis, diabetes, cardiovascular disease (CVD), revision surgeries, and myocardial infarction. Flap loss was linked to increased arteriolar diameters and vein thickness, while preoperative radiotherapy led to a reduced outer diameter of the recipient veins. Alcohol consumption, previous thrombosis, and diabetes were associated with increased total thickness of venous recipient veins, with diabetes also showing venous media dilation. The presence of CVD was related to reduced intimal thickness and increased total vessel wall thickness at the transplant site. Though microvascular reconstruction seems safe even in a complex patient clientele, our findings shed light on the intricate interplay between various factors and vascular parameters, providing valuable insights for clinical practice and further research in reconstructive surgery.

## Figures and Tables

**Table 1:** Clinical characteristics regarding epidemiological and surgical features.

		N = 100	Revisions	*p*-Value	Flap Loss	*p*-Value
Sex	Male	75 (75%)	3		2	
	Female	25 (25%)	3		1	
Age	Years Ø	65 ± 11.1			61 ± 21.4	
Diagnosis				0.01		0.03
	OSCC	63 (63%)	1		-	
	Osteonecrosis of the jaw	17 (17%)	2		2	
	Osteomyelitis	6 (6%)	-		-	
	Cancer of skin	6 (6%)	2		1	
	Other	8 (8%)	1		-	
Flaps				0.9		0.9
	FFF	39 (39%)	3		2	
	RFF	37 (37%)	3		1	
	ALT	18 (18%)	-			
	Scapula	2 (2%)	-			
	Other	4 (4%)	-			
Localization				0.02		0.2
	Mandible	34 (34%)	3		2	
	Floor of the mouth	24 (24%)	0		-	
	Maxilla	14 (14%)	-		-	
	Tongue	9 (9%)	0		-	
	Planum buccale	5 (5%)	2		1	
	Palate	5 (5%)	-		-	
	Other	9 (9%)	1		-	
Flap Revision	Yes	6 (6%)	-		-	
Flap Loss	Yes	3 (3%)	-		-	
Operation time	Min Ø	392 ± 104.2	279 ± 162.8	0.003	221 ± 153.3	0.004
ICU	Days Ø	4 ± 2.7	3.7 ± 1.6	0.9	3 ± 1.7	0.2
NW	Days Ø	19 ± 9	22.4 ± 7	0.7	28 ± 5.1	0.1
Radiation	Yes	66 (66%)	2	0.9	2	0.2
Nicotine	Yes	36 (36%)	1	0.4	2	0.2
Alcohol	Yes	25 (25%)	2	0.6	2	0.1
s.p. Thrombosis	Yes	10 (10%)	1	0.5	-	0.7
s.p. MI	Yes	13 (13%)	1	0.2	-	0.4
CVD	Yes	27 (27%)	1	0.4	-	0.3
Diabetes	Yes	14 (14%)	2	0.3	1	0.2
Recipient artery						
	Facial	58 (58%)	4		2	
	Thyroidal sup.	31 (31%)	2		1	
	Lingual	7 (7%)	-			
	Temporal sup.	4 (4%)	-			
Recipient vein				0.001		0.002
	Facial	44 (47.3%)	4		1	
	Thyroidal sup	33 (35.5%)	-			
	Jugular interna	10 (10.7%)	-			
	Jugular externa	7 (7.5%)	2		2	
	Temporal sup.	4 (4.3%)	-			
	Other	2 (2.2%)	-			

CVD: cardiovascular disease; MI: myocardial infarction; ICU: intensive care unit; NW: normal ward; sup: superior; FFF: free fibula flap; RFF: radial forearm flap; ALT: anterior lateral thigh flap.

**Table 2:** Overall vessel morphology.

	AT	AR	VT	VR
N (191)	70	78	13	30
Diameter Ø	µm	µm	µm	µm
ED	2467.1 ± 549.4	2155.3 ± 515.6	2324.3 ± 683.5	2246.6 ± 663.4
ID	1456.3 ± 408.7	1216.6 ± 410.4	1554.8 ± 601.1	1433.5 ± 617.6
TI	115 ± 74	112 ± 84	32 ± 21	23 ± 16
TM	454 ± 148	407 ± 142	363 ± 160	368 ± 163
TVW	569 ± 181	519 ± 179	395 ± 161	391 ± 170
IMR	0.27 ± 0.19	0.29 ± 0.23	0.1 ± 0.07	0.68 ± 0.03
Hyalinosis	23	21	-	1

ED: external vessel diameter transverse; ID inner vessel diameter transverse; TI: thickness vessel intima; TM: thickness vessel media; TVW: thickness vessel wall; IMR: intima media ratio; AT: artery transplant site; AR: artery recipient site; VT: vein transplant site; VR: vein recipient site; µm: Vessel diameter in micrometers and mean value provided.

**Table 3:** Flap site vessel diameters and associations with epidemiological factors in univariate analysis.

	ATED	ATID	ATTVW	ATTM	ATTI	ATIMR	VTED	VTID	VTTVW	VTTM	VTTI	VTIMR
RFF												
Ø in µm	2604.1	1482.2	642	498	144	0.30	2299.6	1414.8	488	454	34	0.09
Alcohol	2946.6 *p* = 0.003	1690.0 *p* = 0.040	693	544	149	0.29	2381.5	1249.5	602	571	29	0.06
CVD	2550.1	1441.3	671	540	130	0.24	2314.5	1516.5	425	374	54	0.14 *p* = 0.03
ALT												
Ø in µm	2593.3	1468.9	605	495	110	0.22	2730.5	1799.3	332	312	18	0.06
CVD	2656.0	1459.7	710	540	169 *p* = 0.013	0.31 *p* = 0.020						

RFF: radialis forearm flap; ALT: anterior lateral thigh flap; µm: vessel diameter in micrometers and median value provided; CVD: cardiovascular disease.

**Table 4:** Recipient vessel diameters and associations with epidemiological factors in univariate analysis.

	ARED	ARID	ARTVW	ARTM	ARTI	ARIMR
*A. thyroidea* superior						
Ø in µm	2107.7	1197.7	535	413	122	0.32
MI	2584.3	1102.8	925 *p =* 0.003	701 *p =* 0.003	224	0.32
*A. facialis*						
Ø in µm	2216.7	1246.4	511	395	116	0.31
CVD	2255.2	1134.3	584 *p =* 0.037	425	158	0.36
Thrombosis	2616,6 *p* < 0.001	1471.4	672 *p =* 0.016	512 *p =* 0.011	161	0.33
Diabetes	2104.3	1250.6	487	429	59	0.13 *p < 0.001*
Radiation	20589	1149.3 *p =* 0.04	552.8	436.7	114.8 *p =* 0.01	0.30 *p =* 0.02
*A. Temporalis*						
Ø in µm	2067.5	1158	876	790	89	0.11
Radiation	1418.2	862	296.6 *p* = 0.04	235.5 *p* = 0.02	55.5	0.24 *p* = 0.01
	**VRED**	**VRID**	**VRTVW**	**VRTM**	**VRTI**	**VRIMR**
*V. facialis*						
Ø in µm	2360.0	1563.7	388	366	21	0.07
Alcohol	2198.3	1242.8	472 *p* = 0.038	452 *p* = 0.035	20	0.06
Diabetes	2283.5	1394.3	592 *p* = 0.027	577 *p* = 0.022	15	0.02 *p* < 0.001
Radiation	1943.8 *p* = 0.44	1334.8	335	337.5	16.25	0.08
*V. jugularis* interna						
Ø in µm	2012.3	1177.3	383	369	15	0.04
Nicotine	2270.8	1035.8	398	382	20 *p* = 0.002	0.05
CVD	1840	1271.7	373	360	12 *p* = 0.002	0.04

A: artery; V: vein; µm: vessel diameter in micrometers and median value provided; CVD: cardiovascular disease.

**Table 5:** Correlations between vessel diameter and patient characteristics identified using univariate analyses.

Ø in µm		ATID	ATED	ATTM	ARID	ARED	ARTM	ARTVW	VTVW	VTTI	VRID	VRED	VRTM	VRTVW
Sex			*p* = 0.03				*p* = 0.02							
	M		2530.2 ± 28.4				535.45 ± 188.359							
	F		2217.6 ± 533.1				458.33 ± 138.209							
Radiation					*p* = 0.034						*p* = 0.03	*p* = 0.018		
	y				92.25 ± 55						1229.9 ± 341	1966.042 ± 343.3919		
	n				123.38 ± 95.3						1605 ± 716	2494.278 ± 714.0903		
Alcohol			*p* = 0.005	*p* = 0.03										*p* = 0.05
	y		2716.6 ± 372.8	517.28 ± 157.585										519.00 ± 101.628
	n		2363.5± 565.2	431.04 ± 140.707										360.70 ± 169.153
TE							*p* = 0.016	*p* = 0.024						*p* = 0.007
	y						539.75 ± 204.072	657.75 ± 223.713						505.50 ± 149.200
	n						390.76 ± 127.343	502.58 ± 169.848						389.93 ± 169.830
CVD									*p* = 0.017	*p* = 0.016				
	y								396.00 ± 56.107	1355.833 ± 344.4566				
	n								367.10 ± 166.786	1613.850 ± 691.7320				
Diabetes								*p* = 0.009					*p* = 0.007	*p* = 0.002
	y							546.33 ± 178.079					580.50 ± 64.568	476.56 ± 159.931
	n							514.52 ± 181.664					369.50 ± 162.369	396.68 ± 138.723
Revision		*p* = 0.04				*p* = 0.03								
	y	2018.75 ± 35.7				2667 ± 815								
	n	14346 ± 403.5				2119.3 ± 484								
Flap loss					*p* = 0.004	*p* = 0.004								
	y				2012.2 ± 488	3161 ± 847.8								
	n				1188.1 ± 385	2120.7 ± 482								

ATID: artery transplant internal diameter (µm); ATED: artery transplant external diameter (µm); ATTM: artery transplant total media thickness (µm); ARID: artery recipient site internal diameter (µm); ARED: artery recipient vessel external diameter (µm); ARTM: artery recipient site total media thickness (µm); ARTVW: artery recipient site total vessel wall thickness (µm); VTVW: vein transplant total vessel wall thickness (µm); VTTI: vein transplant total intima thickness (µm); VRID: vein recipient site internal diameter (µm); VRED: vein recipient site external diameter (µm); VRTM: vein recipient site total media thickness (µm); VRTVW: vein recipient site total vessel wall thickness (µm); TE: condition after thromboembolism, CVD: cardiovascular disease; µm: vessel diameter in micrometers and mean value provided.

## Data Availability

The data can be obtained by scientists that conduct work independently from the industry, on request. The data are not stored on publicly available servers.
