# Correction: Schuderer et al. Risk Factors for Flap Loss: Analysis of Donor and Recipient Vessel Morphology in Patients Undergoing Microvascular Head and Neck Reconstructions. *J. Clin. Med.* 2023, *12*, 5206

**DOI:** 10.3390/jcm13020548

**Published:** 2024-01-18

**Authors:** Johannes G. Schuderer, Huong T. Dinh, Steffen Spoerl, Jürgen Taxis, Mathias Fiedler, Josef M. Gottsauner, Michael Maurer, Torsten E. Reichert, Johannes K. Meier, Florian Weber, Tobias Ettl

**Affiliations:** 1 Department of Oral and Maxillofacial Surgery, University Hospital Regensburg, 93053 Regensburg, Germany; 2 Institute of Pathology, University Hospital Regensburg, 93053 Regensburg, Germany

In the original publication by Schuderer et al., there was a mistake in Table 1 as published [1]. ‘Radiation’ was falsely 66% instead of 34%. The corrected Table 1 is given below.

There was an error in the original publication. Radiotherapy was falsely described as 66%.

Corrections have been made in the Results section.

In the retrospective patient evaluation, 34% of the patients had previously undergone head and neck radiotherapy, while 36% were documented to have nicotine abuse and 25% had alcohol abuse.

The authors state that the scientific conclusions are unaffected. This correction was approved by the Academic Editor. The original publication has also been updated.

## Figures and Tables

**Table 1:** Clinical characteristics regarding epidemiological and surgical features.

		N = 100	Revisions	*p*-Value	Flap Loss	*p*-Value
Sex	Male	75 (75%)	3		2	
	Female	25 (25%)	3		1	
Age	Years Ø	65 ± 11.1			61 ± 21.4	
Diagnosis				0.01		0.03
	OSCC	63 (63%)	1		-	
	Osteonecrosis of the jaw	17 (17%)	2		2	
	Osteomyelitis	6 (6%)	-		-	
	Cancer of skin	6 (6%)	2		1	
	Other	8 (8%)	1		-	
Flaps				0.9		0.9
	FFF	39 (39%)	3		2	
	RFF	37 (37%)	3		1	
	ALT	18 (18%)	-			
	Scapula	2 (2%)	-			
	Other	4 (4%)	-			
Localization				0.02		0.2
	Mandible	34 (34%)	3		2	
	Floor of the mouth	24 (24%)	0		-	
	Maxilla	14 (14%)	-		-	
	Tongue	9 (9%)	0		-	
	Planum buccale	5 (5%)	2		1	
	Palate	5 (5%)	-		-	
	Other	9 (9%)	1		-	
Flap Revision	Yes	6 (6%)	-		-	
Flap Loss	Yes	3 (3%)	-		-	
Operation time	Min Ø	392 ± 104.2	279 ± 162.8	0.003	221 ± 153.3	0.004
ICU	Days Ø	4 ± 2.7	3.7 ± 1.6	0.9	3 ± 1.7	0.2
NW	Days Ø	19 ± 9	22.4 ± 7	0.7	28 ± 5.1	0.1
Radiation	Yes	34 (34%)	2	0.9	2	0.2
Nicotine	Yes	36 (36%)	1	0.4	2	0.2
Alcohol	Yes	25 (25%)	2	0.6	2	0.1
s.p. Thrombosis	Yes	10 (10%)	1	0.5	-	0.7
s.p. MI	Yes	13 (13%)	1	0.2	-	0.4
CVD	Yes	27 (27%)	1	0.4	-	0.3
Diabetes	Yes	14 (14%)	2	0.3	1	0.2
Recipient artery						
	Facial	58 (58%)	4		2	
	Thyroidal sup.	31 (31%)	2		1	
	Lingual	7 (7%)	-			
	Temporal sup.	4 (4%)	-			
Recipient vein				0.001		0.002
	Facial	44 (47.3%)	4		1	
	Thyroidal sup	33 (35.5%)	-			
	Jugular interna	10 (10.7%)	-			
	Jugular externa	7 (7.5%)	2		2	
	Temporal sup.	4 (4.3%)	-			
	Other	2 (2.2%)	-			

CVD: cardiovascular disease; MI: myocardial infarction; ICU: intensive care unit; NW: normal ward; sup: superior; FFF: free fibula flap; RFF: radial forearm flap; ALT: anterior lateral thigh flap.
